# Investigation on the Achievable Flow Length in Injection Moulding of Polymeric Materials with Dynamic Mould Tempering

**DOI:** 10.1155/2013/845916

**Published:** 2013-07-18

**Authors:** Steve Meister, Dietmar Drummer

**Affiliations:** Friedrich-Alexander-Universität Erlangen-Nürnberg, Institute of Polymer Technology, Am Weichselgarten 9 Tennenlohe, 91054 Erlangen, Germany

## Abstract

A variety of parts in microsystems technology are manufactured by injection moulding of polymeric materials. In Particular the high cooling velocity affects negatively the process and the resulting part properties. The scope of this paper is to investigate the influence on the reachable flow length in injection moulding of different polymeric materials. The results indicate that the mould temperature has less impact on the achievable flow length of the polymer melt as the injection pressure. A higher mould temperature leads only to a slight increase in flow length. In addition, a transcending of the glass or the crystallization temperature of polymeric materials with the mould temperature shows no effect on the achievable flow length of the material.

## 1. Introduction

Microparts and microsystems technology is reputed as a prospective key technology with an estimated annual growth rate of about 10% [1]. The main fields of application of polymer microparts are seen in the areas of medical technology, as components of optical systems, as microgears in microfluidics, biotechnology, and electronics, or as a microelectromechanical system [2, 3]. The demands on the part quality and reproducibility also increase due to the increasing requirements on these microcomponents [4].

A reduction of part dimensions causes an increasing cooling that affects the process and filling behaviour and also the morphological and the mechanical properties of a micropart [5, 6]. In a conventional injection moulding process, the mould surface temperature is far below the melt temperature. This leads to a high cooling velocity and results in a frozen layer close to the mould surface [7]. In addition, the viscosity decreases too which affects the filling behaviour negatively [8]. To counteract this effect, different strategies were developed and investigated to modify and optimize the process parameters. An increasing injection velocity can also favour the transcription of surface structures in the mould [9]. Vetter et al. [10, 11] have investigated an injection moulding process with cavity near melt compression. This allows a ten times higher flow front velocity as in a standard injection moulding process which achieves higher aspect ratios. Also, an increasing pressure [12–14] or a high shear rate [15, 16] can favour the crystallization which is shifted to a higher temperature. Notwithstanding, the most important process parameters that are discussed to influence the cavity filling are the temperatures of the mould and the melt, whereas the mould temperature appears to be the key parameter [8, 17–19]. In general, with increasing mould or melt temperature, the filling behaviour is favoured and an increasing aspect ratio can be reached. In addition, the using of thermal low conductive mould materials [20–22] or a dynamic temperature control of the cavity [23–25] can influence the cooling velocity of the melt. Also, the mould surface roughness can take effect on the mould filling behaviour [26, 27]. Additionally, it was shown that the high cooling velocity affects not only the filling of the cavity but also the morphology (e.g., degree of crystallinity or orientations) and the mechanical properties (e.g., tensile strength) [20, 28–31].

## 2. Experimental

### 2.1. Materials

In the investigations, different thermoplastic polymers were used. A polyamide 66 (PA66, Ultramid A3K, BASF SE), a polypropylene homopolymer (PP, 505P, Sabic Europe), and a polyoxymethylene (POM, Hostaform C9021, Ticona GmbH) as semicrystalline polymers are used. Additionally an amorphous polycarbonate (PC, Makrolon OD2015, Bayer MaterialScience AG) was investigated. Characteristic values of these materials are shown in Table 1. 

These materials were used due to their different crystallization or glass transition temperatures and their different crystallization velocities, respectively.

### 2.2. Specimens

**Figure 1:**
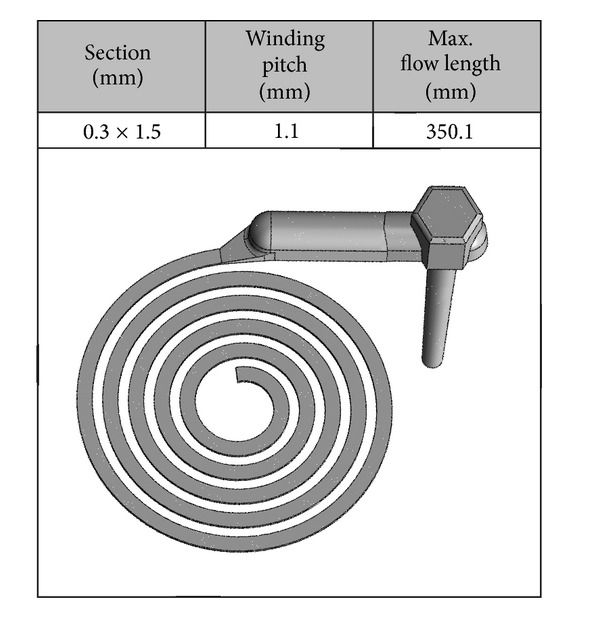
Used microflow spiral for the investigations.

To investigate the influence of mould temperature, a microflow spiral was used, Figure 1. The cross-section of the spiral has a dimension of 0.3 × 1.5 mm. 

### 2.3. Processing

For injection moulding, an Arburg allrounder 370U 700-30/30 injection moulding machine was utilized, equipped with a position controlled screw with a diameter of 15 mm. Relevant process parameters are shown in Table 2. To vary the mould temperature, a variothermal process was implemented. For tempering the mould, a variothermal temperature control system (type: SWTS 200, Single Temperiertechnik GmbH) was used. The system employs water as the circulating fluid and has a heating and a cooling circuit-switching device. It allows a fluid temperature up to 200°C. The mould is maintained at a constant temperature for the purpose of process stability, and only the temperature of cavity inserts is actively controlled. These cavity inserts were built up layer by layer from a steel powder using a rapid tooling process (LaserCusing, Concept Laser GmbH). This manufacturing process allows a complex design of cooling channels, whereby an optimized tempering of the cavity can be performed. The combination of insulation from the master mold and conformal cooling channels conduces to particularly rapid temperature changes in the cavity. The mould temperature is measured by cavity near temperature sensors.

**Figure 2:**
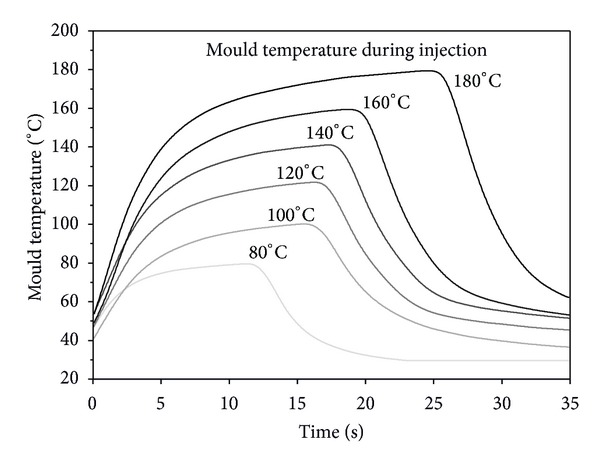
Mould temperature of the variothermal injection moulding process with different mould temperature for injection moulding.

In the investigations, a mould temperature of 80°C up to 180°C was used. After reaching the defined mould temperature, the melt is injected and the mould is cooled down. The curves of the temperature for the different mould temperatures during injection are shown in Figure 2.

As a consequence of an increasing mould temperature, the cooling of the mould after switching to the cold fluid increases too, due to the higher temperature gradient (the cold fluid stays nearly constant). Whilst for a lower mould temperature, the average temperature change is around 12 K s^−1^, and it increases with up to 24 K s^−1^ for a mould temperature of 180°C.

Hence, with the used variothermal tempering process, the temperature of the mould can be above the crystallisation temperature of the PP and the POM or rather above the glass transition temperature of the PC during the injection of the melt. Afterwards, the mould and the melt are cooled down, and a safe ejection of the part can be achieved. For the PA66, the mould temperature is always below the crystallization temperature.

### 2.4. Analytic Approach of the Melt-Mould Contact Temperature

The contact of the polymer melt with the cold mould surface leads to a rapid cooling and solidification of the surface layer of the part. An analytical approach of the contact temperature is shown in [32]. The contact temperature *T*_contact_ is dependent on the temperature of the mould *T*_mould_, the temperature of the polymer melt *T*_polymer_, and the thermal diffusivity *e*:

(1) $$\begin{array}{c} T_{\text{contact}}=\frac{T_{\text{mould}}\cdot e_{\text{mould}}+T_{\text{polymer}}\cdot e_{\text{polymer}}\;}{e_{\text{mould}}+e_{\text{polymer}}\;},\\ e=\sqrt{k\cdot \rho \cdot c_{p}}, \end{array}$$

with *k* the thermal conductivity, *ρ* the density, and the specific heat capacity *c*_*p*_ of the materials. The values for the investigated polymers are shown in Table 1. For the mould material, a density of 7850 kg m^−3^, a thermal conductivity of 29 W m^−1^ K^−1^, and a specific heat capacity of 460 J kg^−1^ K^−1^ were used.

## 3. Results and Discussion

### 3.1. Analytical Calculated Contact Temperature

**Figure 3:**
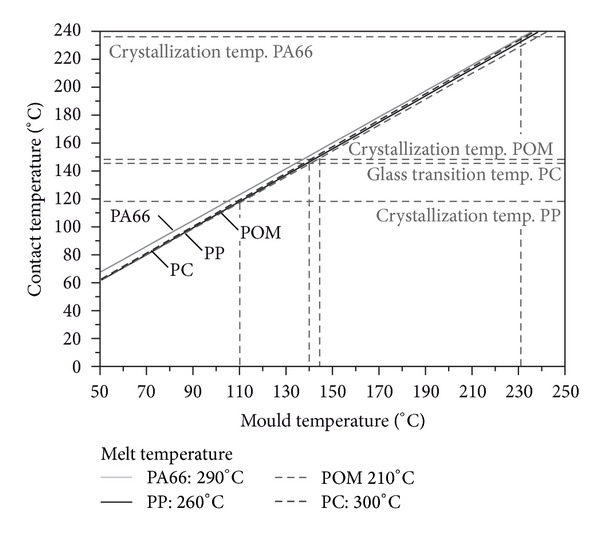
Analytically calculated contact temperature as function of the mould temperature.

Figure 3 shows the analytically calculated contact temperature of the polymers as function of the mould temperature. Due to the high thermal diffusivity of the metal mould, the contact temperature approaches always a marginal higher value as the deployed mould temperature.

Consequently, to achieve a contact temperature above the crystallization temperature for the PP, a mould temperature of about 110°C is needed. For the POM, a mould temperature of about 145°C and for the PA66 ca. 230°C is required. To reach the glass transition temperature of the PC, a mould temperature of about 140°C is needed. As a consequence, the melt flow length should be increased as the glass or crystallization temperature of the polymer material is transcended.

### 3.2. Experimentally Measured Flow Length

**Figure 4:**
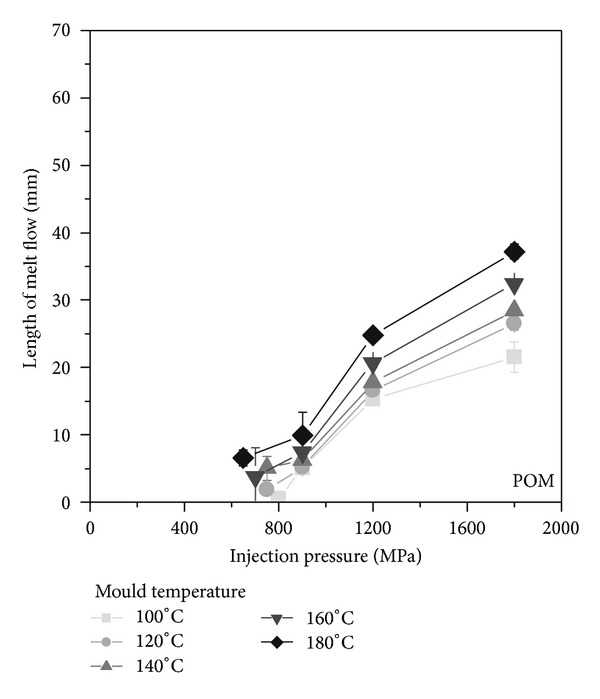
Flow length as function of injection pressure and mould temperature for POM.

Figure 4 shows the flow length for POM as a function of the injection pressure and the varied mould temperature. With the used flow spiral, an injection pressure of about 800 MPa is required to start filling the spiral. The increasing of the mould temperature results in a slight reduction of the required injection pressure. The filling starts at a 25% lower injection pressure but with a limited reliability. In addition, it can be observed that the flow length increases at higher injection pressure as well as at higher mould temperatures. With an injection pressure of 1800 MPa and a mould temperature of 100°C, a flow length of about 21 mm was observed. A mould temperature of 180°C results almost in doubling the flow length to 37 mm. 

Furthermore, with increasing the injection pressure, the flow length of the POM increases with a nonlinear relationship. For an injection pressure of 1200 MPa, it increases in a disproportionately rate but, above, more slowly. This is especially observed for the lower mould temperature, for example, 100°C. It is well known that an increasing pressure affects the crystallization behaviour of semicrystalline polymers [12–14]. This means that in addition to the melt crystallization at the mould due to the cooling, the melt will also solidify as a result of increasing pressure during the filling. As a consequence, the higher injection pressure can lead to a reduced raise of flow length due to the faster crystallization and the higher melt viscosity. Moreover, the results also do not show the expected increase of flow length with transcending the crystallization temperature of the POM. Increasing the mould temperature from 140°C up to 160°C or 180°C does not result in a significant change in the achieved flow length. This means that the flow length is less affected by the increasing mould temperature.

**Figure 5:**
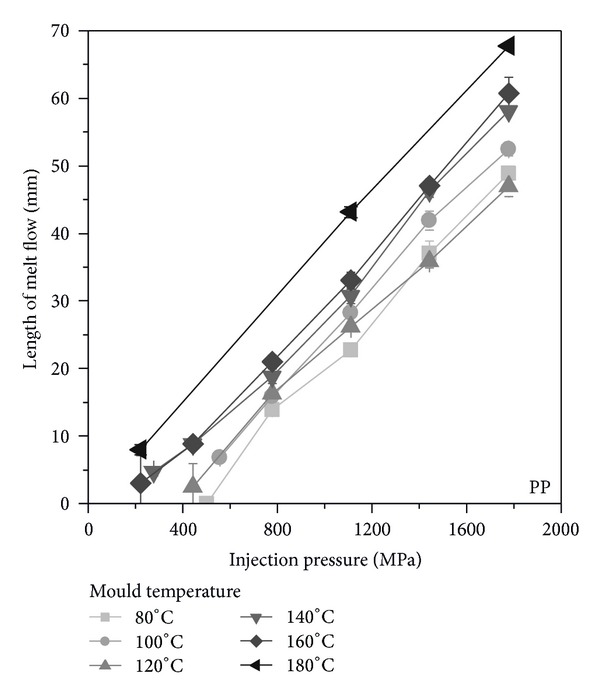
Flow length as function of injection pressure and mould temperature for PP.

With the used polypropylene, the highest flow length has been achieved. The results are shown in Figure 5. The cavity filling starts at a mould temperature of 80°C at 500 MPa and decreases with increasing mould temperature. Using a higher injection pressure leads to increasing the flow length. For each investigated mould temperature, a linear relationship can be observed. That means that for each injection pressure, the flow length for a mould temperature of 80°C and 180°C shows a constant difference of ca. 20 mm. Thus, with an injection pressure of 1800 MPa, the flow length with 80°C is 47 mm and accordingly for 180°C 67 mm. As seen for the POM and for the PP also, no effect on the flow length can be observed, when the mould temperature exceeds the crystallization temperature. 

**Figure 6:**
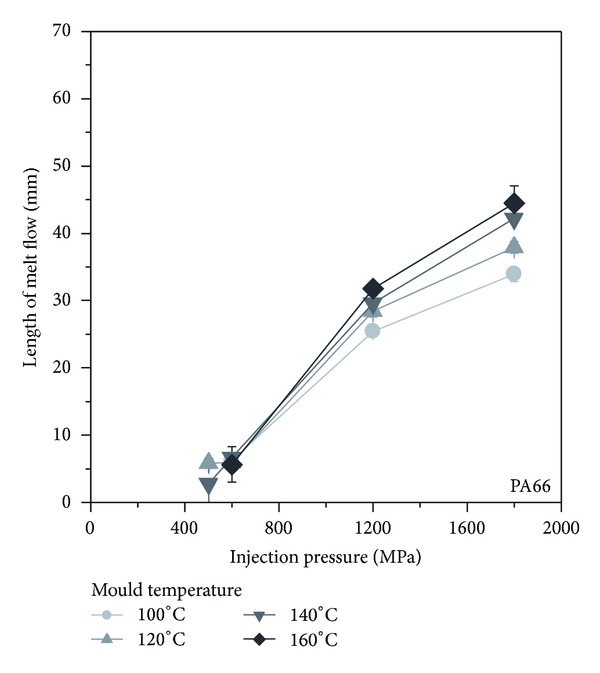
Flow length as function of injection pressure and mould temperature for PA66.

For the PA66, a comparable relationship between injection pressure, mould temperature, and the resulting flow length can be observed as seen for the POM, Figure 6. The flow length increases more to an injection pressure of ca. 120°C, but above the increase is lower. In addition, at a higher injection pressure, it was observed that the mould temperature has more influence on the flow length. While at a lower injection pressure, the mould temperature has no significant influence, neither in starting of cavity filling nor in flow length. At higher injection pressure, the flow length is favoured by an increasing mould temperature.

**Figure 7:**
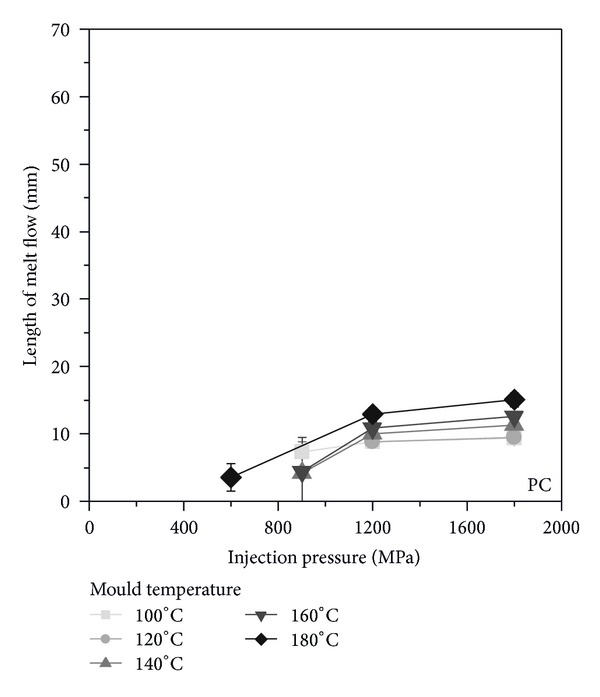
Flow length as function of injection pressure and mould temperature for PC.

The amorphous polycarbonate shows the smallest flow length (Figure 7) due to the high viscosity of the material. The maximum measured flow length was ca. 15 mm. With increasing the injection pressure, the flow length increases to a pressure value of 1200 MPa, and above the flow length remains constant. A higher mould temperature results in a larger flow length, and especially the mould temperature of about 180°C favours noticeably the cavity filling. This is clearly recognizable in the required injection pressure for the beginning of cavity filling. This can be due to a sufficient high mould temperature which is 25°C above the glass transition temperature of the material.

### 3.3. Correlation

**Figure 8:**
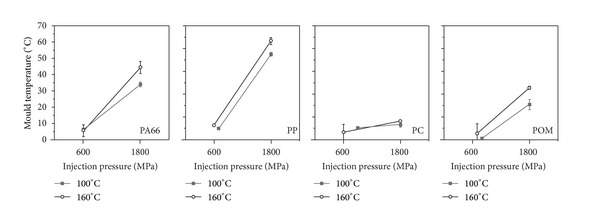
Flow length as function of injection pressure and mould temperature for the different materials.

The effect of injection pressure and mould temperature during injection on the achieved flow length for the investigated materials is shown in Figure 8. For this, the flow length for a mould temperature of 100°C and 160°C and an injection pressure of about 600 MPa (provided that a filling was achieved alternatively the setting was used where a filling occurs) and of 1800 MPa was compared. It is found that the injection pressure has more impact on the attainable flow length as it is found for the mould temperature. A higher mould temperature leads to a slight increase in flow length whose effect is nearly constant with respect to the injection pressure. Only the PA66 shows a marginal disproportional increase of the flow length with higher mould temperature at a high injection pressure.

## 4. Conclusion

In this paper, the influence on the reachable flow length in injection moulding of polymer materials has been investigated. For this, four different polymer materials were used to injection mould a flow spiral as function of injection pressure and mould temperature. To attain a mould temperature significantly above the crystallization or the glass transition temperature, a variothermal process control was used. 

The contact of the hot melt with the colder mould results in a fast cooling. The analytical calculated temperature in the contact area between mould and melt is always near the mould temperature. To prevent a solidification of the melt, the mould temperature must be above the glass or the crystallization temperature of the material to transcend it. 

However, the investigations revealed that the mould temperature has less impact on the achievable flow length of the polymer melt as the injection pressure. A higher mould temperature leads to a slight increase in flow length, but the effect is nearly constant with increasing injection pressure. It was also observed that transcending the glass or the crystallization temperature of the polymer material with the mould temperature has no effect on the achievable flow length of the material. 

The next step in this research is to investigate in detail the crystallization or solidification behaviour of polymer materials with respect to the superposing effects of melt flow and high cooling rates. To carry out measurements, using a rotational viscometer or high-pressure capillary rheometer can lead to more knowledge about the interactions. In addition, the influence of mould temperature and pressure on forming microstructured parts or the effect on mechanical part properties is also a target for further investigations.

## Figures and Tables

**Table 1:** Characteristics of the investigated materials (manufacturer's data).

Parameter	PA66*	PP	POM	PC
Density (*ρ*) (kg·m^−3^)	1130	905	1410	1190
Melting temperature (°C)	260	161	166	—
Crystallization temperature (°C)	236	118	148	—
Glass transition temperature (°C)	90	−20	−70	145
Thermal conductivity (k) (W·m^−1^·K^−1^)	0,33	0,22	0.31	0,2
Specific heat capacity (cp) (J·kg^−1^·K^−1^)	1700	1700	1470	1170

*Dry conditioned.

**Table 2:** Processing parameters

Parameter	PA66	POM	PP	PC
Melt temperature (°C)	290	210	260	300
Injection velocity (cm^3^ s^−1^)	100	100	100	100
